# Safety and efficacy evaluation of low-dose of esketamine combined with propofol for painless gastroscopy: a single-center, randomized, double-blind, parallel controlled clinical trial

**DOI:** 10.3389/fmed.2025.1606134

**Published:** 2025-09-10

**Authors:** Rong-sen Gu, Xiao-Yu Zhuang, Shao-Ping Wu, Xiao-Yu Huang, Zhi-yuan Lin, Yong-Fa Zhang

**Affiliations:** ^1^Department of Anesthesiology, Second Affiliated Hospital of Shantou University Medical College, Shantou, China; ^2^Department of Anesthesiology, Puning People's Hospital, Jieyang, China

**Keywords:** esketamine, propofol, painless gastroscopy, reflex cough, safety

## Abstract

**Background:**

Propofol combined with sufentanil is the most commonly used anesthesia regimen for painless gastroscopy in China. However, this combination carries a higher risk of circulatory and respiratory depression. Esketamine, with its strong analgesic and sympathetic excitatory effects, may be a safer alternative. This study aimed to evaluate the safety and efficacy of propofol-sufentanil versus propofol-esketamine for painless gastroscopy in adults.

**Methods:**

120 participants were randomly assigned to four groups: PS (propofol 2 mg/kg + sufentanil 0.1 μg/kg), PE1 (propofol 2 mg/kg + esketamine 0.05 mg/kg), PE2 (propofol 2 mg/kg + esketamine 0.1 mg/kg), and PE3 (propofol 2 mg/kg + esketamine 0.2 mg/kg). The primary outcome was the incidence of reflex cough during gastroscopy insertion. Secondary outcomes included hemodynamic changes, pulse oxygen saturation, induction time, recovery time, discharge time, propofol consumption, and the occurrence of adverse events.

**Results:**

There was no significant difference in reflex cough, body movement response, or propofol injection pain between the PS, PE2, and PE3 groups, but these incidences were significantly lower than in the PE1 group (*p* < 0.05). Hypotension occurred less frequently in PE2 and PE3 compared to PS and PE1 (*p* = 0.001), with more stable hemodynamics observed in PE2 and PE3. However, the incidence of tachycardia was significantly higher in the PE3 group than in the others (*p* < 0.05). Fewer participants in PE3 and PS required additional propofol compared to PE1 (*p* < 0.05), with no significant difference between PS, PE2, and PE3 (*p* > 0.05). Induction time was significantly shorter in PE3 compared to PS and PE1, with no difference between PE3 and PE2 (*p* > 0.05). However, recovery time was longest in PE3 (*p* = 0.002). No significant differences were found in other outcomes (*p* > 0.05).

**Conclusion:**

Considering the superior safety and efficacy observed in the PE2 group, we recommend the combination of 2 mg/kg propofol and 0.1 mg/kg esketamine as the optimal anesthesia for painless gastroscopy. This combination provides several benefits, including reduced reflex cough, stable hemodynamics, and faster recovery, making it a valuable clinical practice.

## Introduction

Gastroscopy is widely regarded as the gold standard for diagnosing upper gastrointestinal diseases (1). However, as an invasive procedure, it can provoke adverse reactions such as reflexive coughing and vomiting during endoscope insertion, which in turn may increase the risk of respiratory and cardiovascular complications. To reduce these risks and improve patient comfort, many hospitals have adopted the practice of performing gastroscopies under sedation, commonly known as “painless gastroscopy.” This approach aims to alleviate procedural discomfort and minimize the likelihood of adverse effects.

The choice of sedative agents is critical to the success of painless gastroscopy, as the balance between efficacy and safety must be carefully considered. Traditionally, the most commonly used sedative combination for this purpose has been propofol in conjunction with opioids, particularly sufentanil. Propofol is widely utilized due to its rapid onset, quick recovery, and favorable pharmacokinetic profile, which allows for swift induction and recovery from sedation (2). These characteristics make it an attractive option for both anesthesiologists and patients. However, despite its advantages, propofol is not without its complications. Common side effects include pain at the injection site, respiratory depression, and circulatory instability, all of which pose significant risks, especially for patients with underlying health conditions such as cardiovascular or respiratory diseases (3).

To enhance sedation quality and minimize the required dosage of propofol, opioids like sufentanil are often administered concurrently. Sufentanil, a potent opioid, provides effective analgesia and has been shown to reduce the necessary dose of propofol, thus decreasing the incidence of certain side effects. However, opioids, including sufentanil, carry inherent risks such as respiratory depression, apnea, bradycardia, chest wall rigidity, and other gastrointestinal symptoms such as nausea and vomiting. These side effects, particularly respiratory depression, are of significant concern, as they can lead to life-threatening complications during the procedure (4). The safety concerns surrounding the use of propofol and sufentanil in combination have spurred interest in identifying alternative sedative combinations that may offer better safety profiles without compromising the effectiveness of sedation and analgesia.

Esketamine, a dextral isomer of ketamine, has emerged as a promising alternative for sedation and analgesia. Esketamine primarily acts on NMDA (N-methyl-D-aspartate) receptors and provides both sedative and analgesic effects. Research has shown that esketamine’s analgesic potency is twice that of ketamine and four times that of its R-isomer, making it a potent agent for pain management (5). Additionally, esketamine is associated with a lower incidence of adverse effects, particularly those related to psychiatric side effects such as hallucinations and confusion, which are commonly associated with ketamine use. This reduction in psychiatric side effects is due to the absence of R-ketamine in esketamine, as R-ketamine is believed to be responsible for many of the negative mental health effects seen with ketamine (6). However, some studies suggest that high doses of esketamine may still increase the risk of psychiatric side effects (7). Nonetheless, when used in low doses, esketamine has demonstrated a strong analgesic effect with a relatively low risk of these adverse effects (8). In addition to its analgesic properties, esketamine also possesses sympathomimetic effects, meaning it can help stabilize the circulatory system during anesthesia. This is particularly beneficial when used in combination with propofol, as propofol is known to cause respiratory and circulatory suppression. Esketamine’s ability to maintain circulatory stability can counterbalance these effects, providing a more balanced and safer sedation profile during procedures like gastroscopy.

Despite the pharmacological advantages of esketamine, there are still limited clinical reports on its use in combination with propofol for painless gastroscopy. Most of the existing literature focuses on other combinations, such as propofol and sufentanil (9). This gap in the literature underscores the need for further investigation into the potential benefits of combining propofol with esketamine, particularly in the context of painless gastroscopy. Therefore, this study aims to evaluate the safety and efficacy of two sedative combinations—propofol-sufentanil and propofol-esketamine—in adults undergoing painless gastroscopy. Through a comparative analysis, the study seeks to identify a safer and more effective anesthetic regimen that enhances patient comfort while minimizing the risk of adverse effects during the procedure.

## Materials and methods

A total of 120 patients scheduled for painless gastroduodenoscopy at the Endoscopy Center of the Second Affiliated Hospital of Shantou University Medical College between August and December 2022 were included in this study. This was a single-center, prospective, randomized, double-blind trial, approved by the hospital’s Ethics Review Committee (Protocol No. 2022–165) and registered with the Chinese Clinical Trial Center (No. chiCTR 2,300,069,846). All participants provided written informed consent.

### Inclusion and exclusion criteria

The study included 120 patients who met the following criteria: (1) aged 18–64 years; (2) ASA I-II; (3) BMI 18.5–23.9; (4) fully understood the study’s purpose and signed informed consent. Exclusion criteria were: (1) ASA III or higher, or myocardial infarction within the past 6 months; (2) poorly controlled or untreated hypertension; (3) chronic lung diseases or recent acute upper respiratory tract infection or pneumonia; (4) liver or kidney decompensation; (5) neuropsychiatric disorders or epilepsy; (6) anticipated difficult airway; (7) breastfeeding or allergies to eggs, seafood, soy, or medications; (8) history of psychotropic drug or narcotic abuse.

### Randomization and blindness

Patients meeting the inclusion criteria were randomly assigned into four groups using computer-generated randomization. An independent researcher, who was not involved in the sedation process, was responsible for executing the randomization procedure. The anesthesia regimens for each group were as follows: the control group (PS) received 2 mg/kg propofol combined with 0.1 μg/kg sufentanil; trial group 1 (PE1) received 2 mg/kg propofol with 0.05 mg/kg esketamine; trial group 2 (PE2) received 2 mg/kg propofol with 0.1 mg/kg esketamine; and trial group 3 (PE3) received 2 mg/kg propofol with 0.2 mg/kg esketamine. The drugs were prepared by a researcher not involved in the sedation process and administered by an anesthesiologist who was blinded to the group assignments. Data collection was carried out by a separate researcher who was also blinded to the group allocations. To ensure blinding, both the anesthesiologists and data collectors, as well as the endoscopists and patients, remained unaware of the group assignments throughout the study.

### Sample size estimation

The sample size for this study was determined through a preliminary pre-test, which indicated the following efficacy in preventing reflex cough during gastroscopy insertion: 80, 50, 80, and 90% for the PS, PE1, PE2, and PE3 groups, respectively. A power analysis was performed with a significance level (*α*) of 0.05 and a desired power (*β*) of 0.80, considering a 15% anticipated loss to follow-up. Based on these parameters, the calculated sample size was 120 participants, with 30 participants per group.

### Anesthesia sedation process

The day before the gastroscopy, the anesthesiologist should conduct a routine visit, recording the patient’s basic information such as age, gender, height, weight, body mass index (BMI), medical history, anesthesia contraindications, and ASA classification. For any potential conditions that may affect anesthesia safety, relevant tests should be arranged. For patients meeting the trial inclusion criteria, the anesthesia and endoscopy process, along with the potential benefits and risks of participating in the trial, should be explained in detail to the patient or their immediate family. After obtaining informed consent from the patient or family member, both the anesthesia consent and the clinical trial consent should be signed, and the patient can then be included in the trial.

A researcher not involved in anesthesia or data collection diluted the required drugs to a final volume of 10 mL using normal saline, resulting in sufentanil at 1 μg/mL and esketamine at concentrations of 0.5 mg/mL, 1 mg/mL, and 2 mg/mL. The propofol stock solution had a concentration of 10 mg/mL and was used directly without dilution.

All patients fasted for at least 8 h and refrained from drinking for at least 2 h before the gastroscopy. Fifteen minutes before the procedure, they consumed about 100 mL of a vesicle removal solution (streptomycin granules, simethicone emulsion, sodium bicarbonate, and water) prepared by the endoscopic nurse. Upon entering the room, patients were positioned on their left side, and intravenous access was established in the right upper limb. Sodium potassium magnesium calcium and glucose injection were administered, and low-flow oxygen (2 L/min) was delivered via a nasal catheter. Routine monitoring included ECG, pulse oximetry, and left upper limb arterial blood pressure.

Patients received intravenous injections of specific doses of sufentanil or esketamine, followed by a 2 mg/kg dose of propofol. Anesthesia induction was monitored by recording the time from the end of drug administration to the disappearance of the eyelash reflex. Sedation depth was assessed using the Ramsay Sedation Scale (RSS), with a target RSS score of ≥5 indicating adequate sedation. If the RSS score was <5, an additional 0.5 mg/kg of propofol was administered. Endoscopy was initiated once the RSS reached ≥5. During the procedure, the anesthesiologist continuously monitored the patient, administering 0.5 mg/kg doses of propofol as needed to maintain an RSS score of ≥5, ensuring proper sedation and patient safety. The doses of propofol, sufentanil, and esketamine used during anesthesia were also recorded. Blood pressure (BP), heart rate (HR), and pulse oxygen saturation (SpO_2_) were measured at five time points: T0 (upon entering the room), T1 (when eyelash reflex ceased), T2 (when the gastroscope was inserted), T3 (at the end of the procedure), and T4 (5 min after the procedure).

Hypotension was defined as a systolic blood pressure below 90 mmHg or a decrease of more than 30% from baseline; hypertension as an increase of more than 30% from baseline; and hypoxemia as an SpO2 below 94%. Bradycardia was defined as a heart rate below 60 beats per minute, and tachycardia as a heart rate above 100 beats per minute.

If the patient experiences hypotension during the examination, fluid rehydration should be increased, and 10 mg of intravenous ephedrine should be administered. If bradycardia occurs, 0.5 mg of atropine should be injected intravenously. In the case of hypertension, sedation depth should be adjusted accordingly, and the procedure should be paused if necessary. If hypoxemia occurs, increase oxygen flow and support the jaw to open the airway. If blood oxygen levels cannot be restored, insert a nasopharyngeal airway and suspend the endoscopy. A simple balloon mask can be used to administer oxygen if needed. If the patient coughs during endoscope insertion, additional anesthesia should be given. In the event of serious adverse effects, such as severe coughing that interferes with the procedure or anaphylactic shock, the trial should be immediately suspended, and blinding measures should be implemented to ensure the patient’s safety.

### Primary and secondary outcome

The primary outcome of this study is the incidence of reflex cough following gastroscopy insertion. Secondary outcomes include hemodynamic indices (T0-T4), induction time, recovery time (from the end of examination to when the patient’s Ramsay score reaches 2), discharge time (from recovery time to when the PADSS score is ≥9), propofol consumption, patient and endoscopist satisfaction, and adverse events. Adverse events include pain from propofol injection, hypoxemia, hypotension, hypertension, arrhythmia, delirium, lethargy, nausea and vomiting, body movement, apnea, excessive oral secretions, and psychotomimetic effects.

### Statistical analysis

Data collection adhered to the protocols of the prospective intervention study. Statistical analysis was conducted using SPSS 25.0. Continuous variables were presented as mean ± SD, and categorical variables as *N* (%). Before analysis, the Kolmogorov–Smirnov test and histograms were used to assess data normality. For continuous variables, analysis of variance (ANOVA) was performed, with *post hoc* comparisons conducted using the Student–Newman–Keuls (SNK) test. Data collected through repeated measures were analyzed using a repeated-measures ANOVA. Categorical variables were assessed using either the chi-square test or Fisher’s exact test. A *p*-value less than 0.05 was considered statistically significant.

## Results

A total of 120 participants who met the trial inclusion criteria were enrolled. No participants were withdrawn due to adverse events during the examination. Follow-up was conducted by telephone 1 day after gastroscopy, with no participants lost to follow-up. Therefore, all 120 patients successfully completed the trial and were included in the data analysis.

### General information

No significant differences were observed in age, sex, BMI, body weight, ASA grade, or other variables among the four groups (*p* > 0.05) (Table 1).

**Table 1:** General information of individuals.

General information	Group PS	Group PE1	Group PE2	Group PE3	*p* value
Gender (male/female)	13/17	13/17	10/20	13/17	0.818
ASA (I/II)	21/9	22/8	23/7	22/8	0.952
Age (years)	40.7 ± 11.5	41.6 ± 10.0	36.8 ± 8.1	37.5 ± 8.2	0.054
Height (cm)	163 [157.7, 171]	160 [158, 171.25]	161.5 [158.75, 169.25]	162 [158.75, 170]	0.997
Weight (kg)	58.5 [50, 64.3]	57.5 [49.8, 66.5]	55 [50, 63.5]	57.3 [50, 61.3]	0.186
BMI (kg/m^2^)	21.77 [19.02, 23.28]	21.06 [19.61, 23.67]	21.05 [19.64, 23.4]	20.80 [19.4, 22.79]	0.960
Smoking (yes/no)	4/26	2/28	2/28	3/27	0.900
Drink (yes/no)	2/28	0/30	2/28	1/29	0.753
Hypertension (yes/no)	4/26	1/29	2/28	2/28	0.625
Diabetes (yes/no)	0/30	1/29	0/30	0/30	1.000
COPD (yes/no)	0/30	0/30	0/30	0/30	1.000
Asthma (yes/no)	0/30	0/30	0/30	0/30	1.000

### Incidence of adverse effects

**Figure 1:**
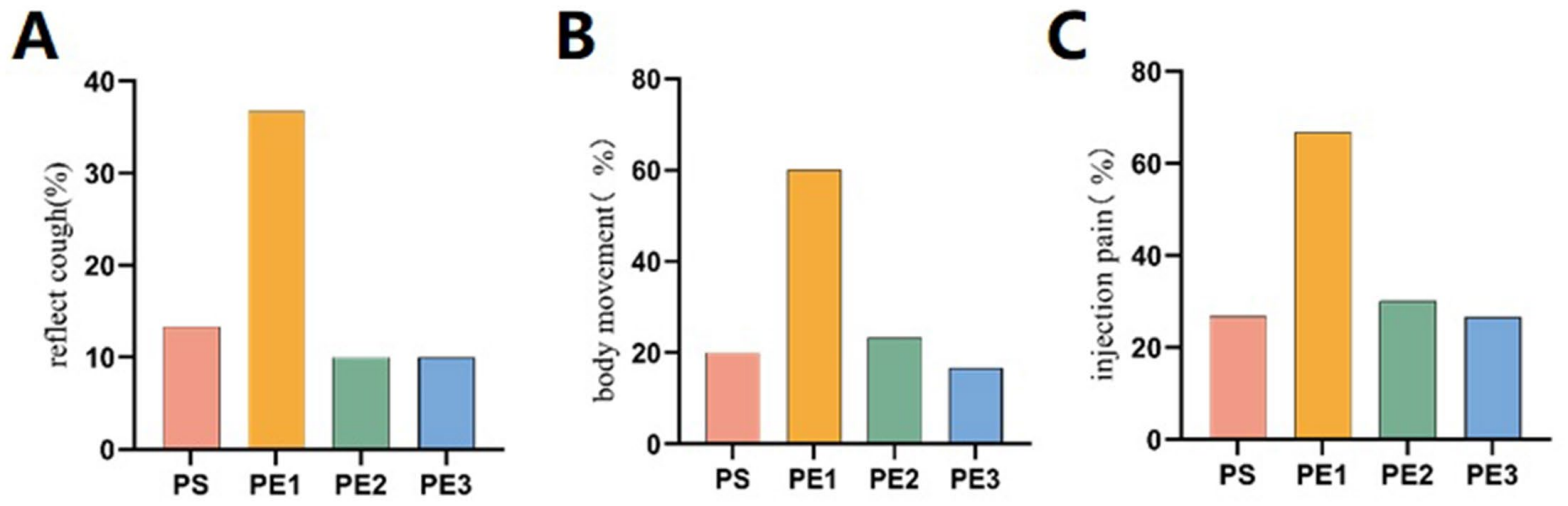
Comparison of the incidence of reflex cough, body movement, and propofol injection pain among the groups. **(A)** The incidence of reflex cough was significantly higher in the PE1 group compared to the PS, PE2, and PE3 groups (*p* < 0.05). There were no significant differences in reflex cough incidence among the other three groups (*p* > 0.05). **(B)** The incidence of body movement during examination differed significantly among the four groups (*p* = 0.001). The PE1 group had a significantly higher incidence than the PS, PE2, and PE3 groups (*p* < 0.05). There were no significant differences among the other three groups. **(C)** The incidence of propofol injection pain differed significantly among the four groups (*p* = 0.002), with the PE1 group showing a significantly higher incidence. No significant differences were found between the PS, PE2, and PE3 groups (*p* > 0.05).

The incidence of adverse effects in the four groups during and within 24 h after the examination was presented (Table 2). Significant differences were observed among the groups for reflex cough (*p* = 0.029) (Figure 1A), body movement (*p* = 0.001) (Figure 1B), propofol injection pain (*p* = 0.002) (Figure 1C), tachycardia (*p* = 0.048), hypotension (*p* = 0.001), and drowsiness (*p* = 0.001). The incidence of drowsiness in group PS was significantly higher than in groups PE1 and PE2 within 24 h after the examination, but there was no significant difference compared with group PE3.

**Table 2:** Incidence of adverse effects.

Adverse effects	Group PS	Group PE1	Group PE2	Group PE3	*p* value
Cough	4 (13.3%)^a^	11 (36.7%)^b^	3 (10%)^a^	3 (10%)^a^	0.029
Body movement	6 (20%)^a^	18 (60%)^b^	7 (23.3%)^a^	5 (16.7%)^a^	0.001
Injection pain	8 (26.7%)^a^	20 (66.7%)^b^	9 (30%)^a^	8 (26.7%)^a^	0.002
Hypoxemia	1 (3.3%)	1 (3.3%)	0 (0)	1 (3.3%)	1.000
Tachycardia	1 (3.3%)^a^	5 (16.7%)^a,b^	4 (13.3%)^a,b^	9 (30%)^b^	0.048
Bradycardia	9 (30%)	2 (6.7%)	4 (13.3%)	3 (10%)	0.095
Hypotension	9 (30%) ^a^	6 (20%)^a,b^	1 (3.3%)^b^	0 (0)^b^	0.001
Hypertension	0 (0)	0 (0)	0 (0)	0 (0)	1.000
Delirium	0 (0)	0 (0)	0 (0)	0 (0)	1.000
Drowsiness	12 (40%)^a^	2 (6.7%)^b^	2 (6.7%)^b^	5 (16.7%)^a,b^	0.001
Dizzy	9 (30%)	12 (40%)	11 (36.6%)	12 (40%)	0.835
Nausea	1 (3.3%)	0 (0)	1 (3.3%)	0 (0)	1.000
Emesis	0 (0)	0 (0)	0 (0)	0 (0)	1.000
Visual disturbance	0 (0)	0 (0)	1 (3.3%)	1 (3.3%)	1.000
Psychoactive effects	0 (0)	0 (0)	0 (0)	0 (0)	1.000

“ab” indicates the difference in diastolic blood pressure between the four groups at the same time. Groups sharing the same letter have no statistically significant difference (*p* > 0.05), while groups with different letters show a statistically significant difference (*p* < 0.05).

### Vital signs

**Figure 2:**
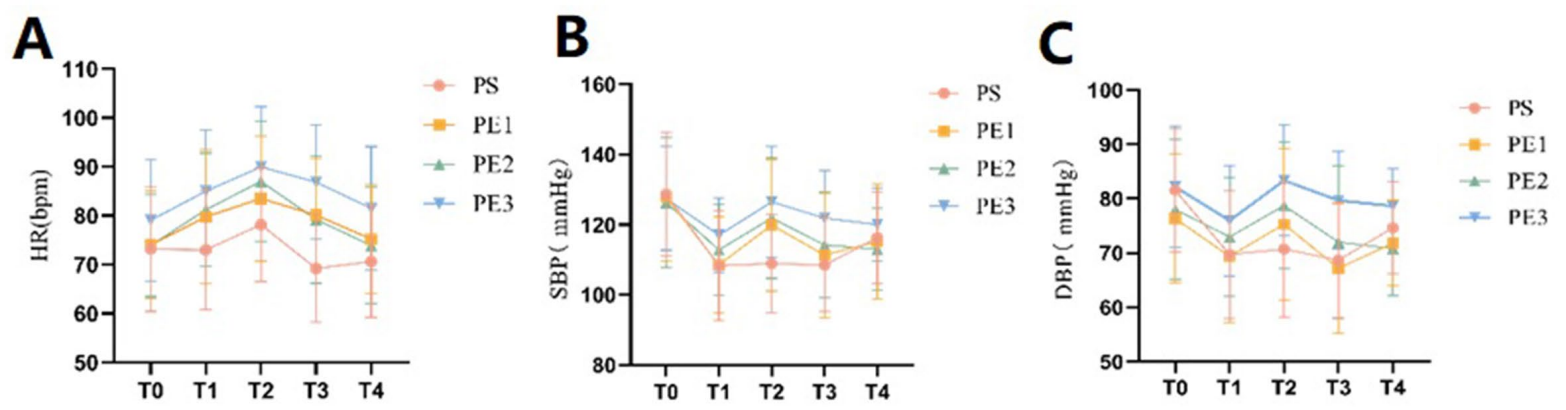
Comparison of vital sign parameters of the four groups at different time points. **(A–C)** The incidence of hypotension differed significantly among the four groups (*p* = 0.001). The PE2 and PE3 groups had a significantly lower incidence than the PS and PE1 groups. Systolic and diastolic blood pressure, as well as heart rate, remained stable in the PE2 and PE3 groups during T1, T2, and T3 compared to the PS and PE1 groups. However, the tachycardia rate was significantly higher in the PE3 group than in the other groups (*p* < 0.05).

The vital parameters included systolic blood pressure (SBP), diastolic blood pressure (DBP), HR, and SpO2, measured at five time points: T0, T1, T2, T3, and T4 (Supplementary Tables S1–S4). Changes in these parameters for the four groups at each time point were shown (Figures 2A–C). There were no significant differences in vital signs among the four groups at T0 (*p* > 0.05). However, significant differences in HR, SBP, and DBP were observed at T1, T2, T3, and T4 (*p* < 0.05). No significant differences were found in SpO2 across the groups at the five time points (*p* > 0.05). The incidence of hypotension was significantly lower in groups PE2 and PE3 compared to group PS, with no significant difference between group PS and group PE1. The incidence of hypotension was 20, 3.3, and 0% in groups PE1, PE2, and PE3, respectively. While no statistical difference was observed among the three groups, a dose-dependent decreasing trend was noted.

### Time index

**Figure 3:**
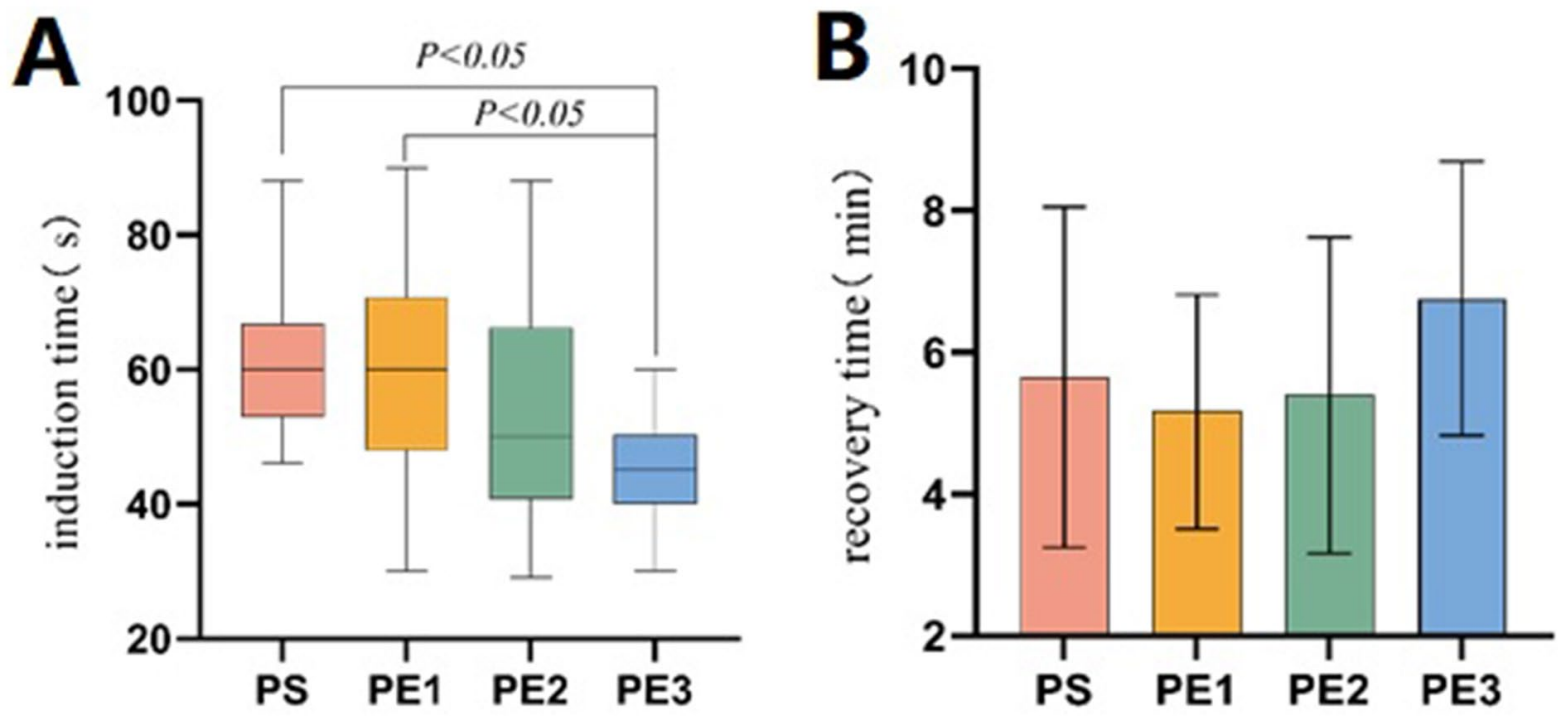
Comparison of induction and recovery times among the groups. **(A)** The induction time differed significantly among the four groups (*p* < 0.001). The induction time in the PE3 group was significantly shorter than in the PS and PE1 groups (*p* < 0.05), but there was no significant difference compared to the PE2 group (*p* > 0.05). **(B)** The recovery time differed significantly among the four groups (*p* = 0.002). The recovery time in the PE3 group was significantly longer than in the other three groups (*p* < 0.05). No significant difference was found between the PS, PE1, and PE2 groups (*p* > 0.05).

The statistics of induction, procedure, recovery, and discharge times among the four groups were presented (Table 3). The induction time in group PE3 was significantly shorter than in groups PS and PE1, but no significant difference was found between group PE2 and group PS (Figure 3A). The recovery time in group PE3 was significantly longer than in the other three groups, with no significant differences among the other three groups (Figure 3B). There were no significant differences in procedure time and discharge time among the four groups.

**Table 3:** Time index.

Time index	Group PS	Group PE1	Group PE2	Group PE3	*p* value
Induction time	60 [53,66.75]^a^	60 [48,70.75] ^a^	50 [40.75,66.25] ^a,b^	45 [40,50.25]^b^	<0.001
Procedure time	5.78 ± 0.59	5.67 ± 0.60	5.44 ± 0.81	5.50 ± 0.73	0.256
Recovery time	5.52 ± 1.76^a^	5.16 ± 1.66^a^	5.40 ± 2.23^a^	6.89 ± 1.75^b^	0.002
Discharge time	12.63 ± 2.96	11.41 ± 2.86	12.71 ± 2.28	12.83 ± 2.11	0.121

“ab” indicates the difference in diastolic blood pressure between the four groups at the same time. Groups sharing the same letter have no statistically significant difference (*p* > 0.05), while groups with different letters show a statistically significant difference (*p* < 0.05).

### Propofol consumption

**Figure 4:**
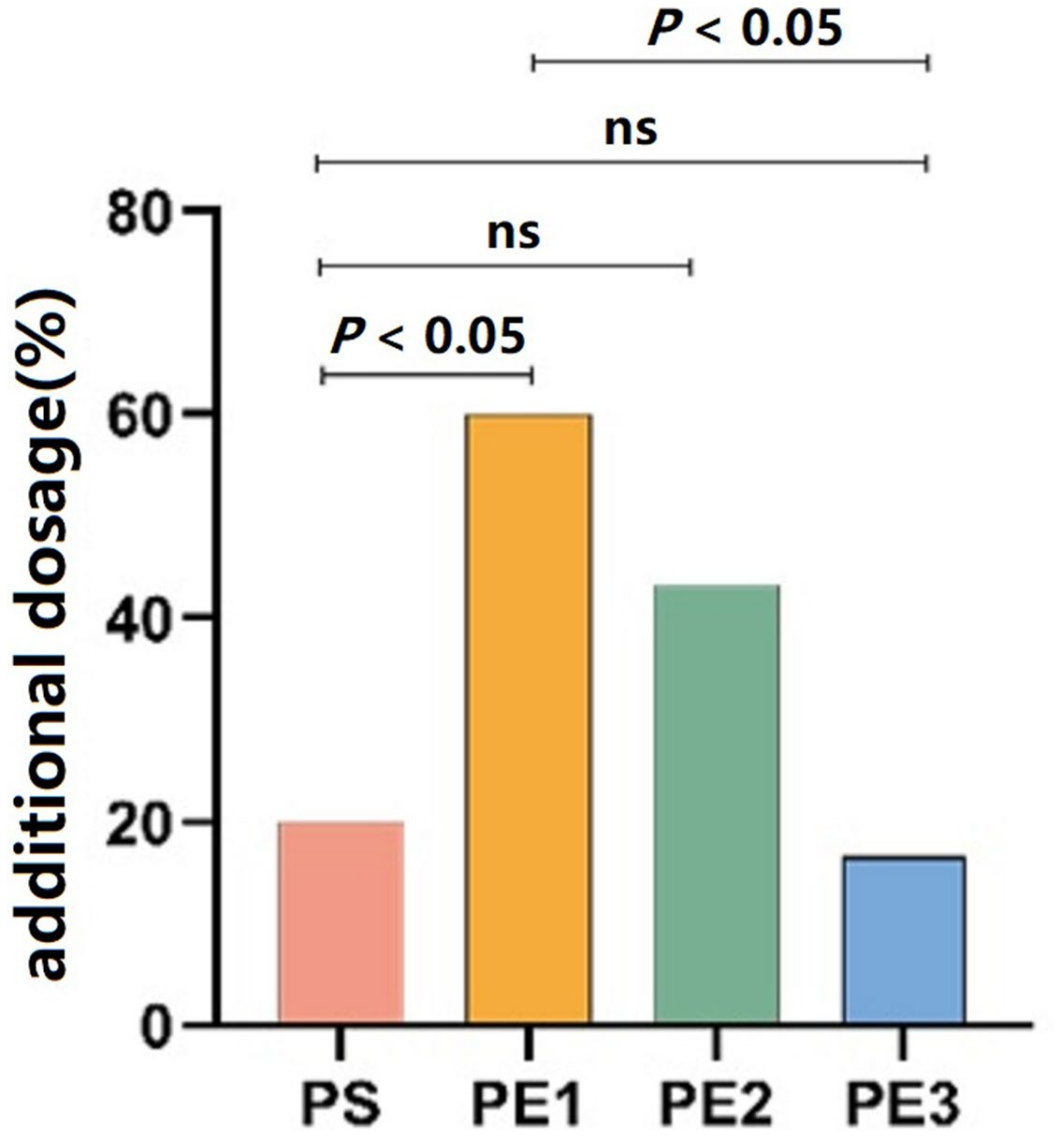
Comparison of propofol consumption across the groups. The number of patients requiring additional propofol was significantly lower in the PE3 and PS groups compared to the PE1 group (*p* < 0.05), but there was no significant difference between the PS, PE2, and PE3 groups.

Patients in groups PS, PE1, PE2, and PE3 who required additional propofol only needed one dose to meet the examination needs, with numbers of 6, 18, 13, and 5, respectively (*p* = 0.001). The need for additional propofol was significantly lower in groups PE3 and PS compared to group PE1 (*p* < 0.05). However, there was no statistically significant difference in the need for additional propofol between the PS, PE2, and PE3 groups (Figure 4).

## Discussion

Reflex cough is a common complication of gastroscopy that causes discomfort for patients, affects their physiology, and interferes with the procedure and observation for endoscopists. Evaluating whether a painless anesthesia or sedation regimen offers advantages in effectively suppressing reflex cough is crucial. Coughing is a protective airway reflex to expel foreign objects from the respiratory tract, and it can be classified into voluntary, induced, and reflex coughs (10). The main difference between induced and reflex coughs lies in the intensity of the stimulation. When the stimulation reaches a certain threshold, induced cough can turn into reflex cough. Reflex cough can be triggered by irritation during gastroscopy placement or from oropharyngeal secretions. While propofol can inhibit upper respiratory responses and raise the threshold for reflex cough, the strong pharyngeal stimulation caused by gastroscopy can still provoke reflex coughing (11). Severe coughing increases abdominal pressure and activates the sympathetic nervous system, leading to significant hemodynamic fluctuations in patients (11). To minimize these adverse effects, propofol is often combined with other anesthetics such as sufentanil, midazolam, etomidate, or ketamine. However, no sedation regimen has yet been developed that fully satisfies patients, endoscopists, and anesthesiologists.

Esketamine is an NMDA receptor antagonist with stronger sedative effects. Due to the dose-dependent side effects of ketamine, low-dose esketamine can reduce the incidence of anesthetic side effects. Studies have shown that low-dose esketamine helps reduce adverse events, such as reflex cough and hypotension, by stimulating the sympathetic nervous system, providing analgesia, and antagonizing NMDA receptors (12). In a clinical trial comparing the incidence of reflex cough during painless gastroscopy, propofol combined with saline, sufentanil, or ketamine resulted in reflex cough rates of 40, 17, and 3%, respectively (13). The ketamine dose in this study was 0.4 mg/kg, which is equivalent to 0.2 mg/kg of esketamine based on titer calculations. Our study found that doses of esketamine at 0.1 mg/kg or higher effectively inhibited reflex cough. However, 0.05 mg/kg of esketamine did not significantly reduce the occurrence of reflex cough, likely due to insufficient analgesia at this lower dose.

Propofol combined with sufentanil can easily cause hypotension after anesthesia induction (14). Some studies suggest that this is due to arterial dilation caused by propofol, leading to reduced systemic vascular resistance, rather than venous dilation or changes in myocardial contractility (15). Esketamine has a sympathetic-like effect, which constricts resistant blood vessels and partially alleviates the cardiovascular depression caused by propofol. Zhou et al. (16) reported that 0.5 mg/kg of esketamine could effectively improve peripheral perfusion and mean arterial pressure after anesthesia induction. In our study, we also found that the sufentanil group experienced a more significant decrease in blood pressure compared to the esketamine groups, with the incidence of hypotension being dose-dependent on esketamine. Therefore, we speculate that esketamine’s protective effect against hypotension may be dose-dependent. However, further clinical studies with larger sample sizes are needed.

Literature indicates that esketamine doses below 0.25 mg/kg primarily produce an analgesic effect, with 0.125–0.25 mg/kg having additional sedative effects (17, 18). Studies have also shown that combining 1.5 mg/kg propofol with 0.2 mg/kg esketamine reduces induction time in painless gastroenteroscopy, suggesting that 0.2 mg/kg esketamine may enhance the sedative effect of propofol (19). In our study, we found that 0.2 mg/kg or higher doses of esketamine shortened the induction time, consistent with previous research. However, there are some discrepancies when comparing our findings to those of Zhang et al. (20), who reported that 0.2 mg/kg esketamine prolonged recovery time after general anesthesia. Similarly, Feng et al. (21) found that 0.5 mg/kg esketamine significantly prolonged recovery time in a dose-dependent manner compared to 0.15 mg/kg and 0.25 mg/kg doses during painless gastroenteroscopy. In contrast, our study did not observe a prolongation of recovery time with doses below 0.2 mg/kg, while doses of 0.2 mg/kg or higher appeared to extend recovery time. The differences in our findings may result from variations in study design, patient populations, and procedures. The combination of esketamine with other sedatives, along with the specific clinical context, could also influence recovery outcomes. Further research is needed to clarify how esketamine dosage affects both induction and recovery phases.

The optimal dosage of esketamine for painless gastroenteroscopy remains unclear. It has been reported that 0.5 mg/kg esketamine alone is safe and effective for gastroscopy in Chinese patients (12). However, most clinical anesthesiologists believe this dose may be linked to a higher risk of side effects, such as increased secretions and adverse mental effects. Low-dose esketamine primarily serves an analgesic role and is often combined with propofol for anesthesia, reducing the need for higher doses of propofol (22). In our study, we found that as the combined esketamine dose increased, the number of patients requiring additional propofol decreased. This suggests that esketamine may reduce propofol use in painless gastroenteroscopy in a dose-dependent manner.

Visual disturbances are a common adverse effect of esketamine. Previous studies have shown that esketamine may increase the incidence of visual impairment in a dose-dependent manner, with high-dose administration being a primary cause of prolonged recovery times and poorer recovery quality in patients (23, 24). Notably, in our study, only one patient in the PE3 group experienced transient visual impairment, characterized by blurred vision that resolved within 1 h, while no visual impairment was observed in the other groups. Based on these findings, we hypothesize that low-dose esketamine, as used in painless gastroscopy, does not significantly increase the risk of visual impairment. However, we emphasize the importance of remaining vigilant for this potential adverse effect in clinical practice.

Drowsiness is a common adverse effect associated with opioids (25). In this study, we found that low doses of esketamine did not significantly increase the incidence of drowsiness after the procedure, whereas high doses of esketamine and sufentanil exhibited similar potential to induce drowsiness in patients. Importantly, no other adverse mental effects were observed in our investigation. Several factors may explain these findings: First, esketamine lacks R-ketamine, which is believed to cause adverse mental effects (26). Second, mental side effects are mainly dose-dependent, and the low dose of esketamine used in this study likely contributed to the absence of such effects. Third, propofol can mitigate the psychogenic side effects of esketamine by inhibiting c-fos expression in the cingulate cortex, a mechanism linked to ketamine-induced mental side effects (27). Finally, esketamine can help alleviate cognitive dysfunction and brain damage caused by propofol (28). Based on these factors and our results, we conclude that low-dose esketamine combined with propofol for painless gastroscopy may not increase the incidence of psychiatric side effects.

Opioids and propofol can both cause respiratory depression (29). Ketamine can reduce ventilation insufficiency by stimulating the sympathetic nervous system, which helps maintain spontaneous breathing and airway reflexes (30). In our study, hypoxemia occurred infrequently in both the sufentanil and esketamine groups. We attribute this to two factors. First, the patients in our study were classified as ASA I or II, excluding those who are overweight or obese, as these individuals are more susceptible to respiratory insufficiency during induction. Second, all patients received a low-flow (2 L/min) nasal oxygen catheter, which effectively mitigated respiratory depression caused by propofol and sufentanil. Consequently, none of the four anesthesia regimens used in this study resulted in significant respiratory depression during painless gastroscopy.

This study compared the safety and efficacy of different doses of esketamine combined with propofol for painless gastroscopy, aiming to provide new insights into esketamine’s application. However, several limitations should be addressed in future research. First, the age range of 18 to 64 years may not fully represent older populations, and future studies should expand the sample size and consider age stratification. Second, manual intravenous administration could lead to dosing inaccuracies compared to more precise methods like target-controlled infusion (TCI). Incorporating TCI with real-time bispectral index (BIS) monitoring would improve dosing accuracy and sedation control. Lastly, variability in technique among gastroscopic operators could have contributed to inconsistent propofol administration. Future studies should standardize operator training or use a consistent group of operators to minimize this bias and improve procedural uniformity.

## Conclusion

The combination of 0.1 mg/kg esketamine and 2 mg/kg propofol demonstrates a favorable balance of safety and efficacy for painless gastroscopy. This regimen is associated with a low incidence of reflex cough, stable hemodynamics, and no significant effect on recovery time or major adverse events, making it a promising anesthetic option for such procedures. Future studies should examine the effects of different esketamine doses to optimize safety and effectiveness. Long-term research is also necessary to evaluate the sustained safety and efficacy of this anesthetic combination across diverse clinical settings.

## Data Availability

The original contributions presented in the study are included in the article/Supplementary material, further inquiries can be directed to the corresponding author.
