# Supplementary material for: Safety and efficacy evaluation of low-dose of esketamine combined with propofol for painless gastroscopy: a single-center, randomized, double-blind, parallel controlled clinical trial
Source: Front Med (Lausanne). 2025 Sep 10;12:1606134. doi: 10.3389/fmed.2025.1606134 (PMC12457402; doi:10.3389/fmed.2025.1606134)
Supplement: Supplementary file 1 [file Table_1.PDF]

Table S1. Patient's heart rate (HR) at different times (bpm)

	Group PS	Group PE1	Group PE2	Group PE3	*P**
T0	73.17±12.75	74.03±10.99	73.93±10.47	79.10±12.43	0.188
T1	73.00±12.15^{a}	79.80±12.78$^{a, b}$	81.23±11.62^{b}	85.00±12.60^{b}	0.003
T2	78.17±11.65^{a}	83.53±13.62$^{a, b}$	87.00±12.3^{b}	89.97±12.90^{b}	0.002
T3	69.20±11.00^{a}	80.13±11.54^{b}	79.23±13.02^{b}	86.67±11.63^{b}	<0.001
T4	70.63±11.45^{a}	75.23±11.21$^{a, b}$	73.90±11.90$^{a, b}$	81.53±12.61^{b}	<0.001

"ab" indicates the difference in diastolic blood pressure between the four groups at the same time. Groups sharing the same letter have no statistically significant difference ($P > 0.05$), while groups with different letters show a statistically significant difference ($P < 0.05$).
