# Supplementary material for: Safety and efficacy evaluation of low-dose of esketamine combined with propofol for painless gastroscopy: a single-center, randomized, double-blind, parallel controlled clinical trial
Source: Front Med (Lausanne). 2025 Sep 10;12:1606134. doi: 10.3389/fmed.2025.1606134 (PMC12457402; doi:10.3389/fmed.2025.1606134)
Supplement: Supplementary file 2 [file Table_2.PDF]

Table S2. Patient's systolic blood pressure (SBP) at different times (mmHg)

	Group PS	Group PE1	Group PE2	Group PE3	*P**
T0	128.67±17.65	127.90±18.33	126.33±18.54	127.53±14.82	0.963
T1	107.27±15.95[a]	108.57±13.83[a, b]	112.83±12.93[a, b]	117.03±10.62[b]	0.024
T2	108.97±14.33[a]	119.87±18.70[a, b]	121.90±17.12[b]	126.53±15.75[b]	0.001
T3	108.53±13.22[a]	111.37±17.91[a]	114.20±14.92[a, b]	121.87±13.50[b]	0.006
T4	116.30±13.00	115.20±16.33	113.00±11.72	120.03±10.37	0.212
P(T1vsT0)	<0.001	<0.001	<0.001	<0.001	

"ab" indicates the difference in diastolic blood pressure between the four groups at the same time. Groups sharing the same letter have no statistically significant difference ($P > 0.05$), while groups with different letters show a statistically significant difference ($P < 0.05$).
