# Supplementary material for: Safety and efficacy evaluation of low-dose of esketamine combined with propofol for painless gastroscopy: a single-center, randomized, double-blind, parallel controlled clinical trial
Source: Front Med (Lausanne). 2025 Sep 10;12:1606134. doi: 10.3389/fmed.2025.1606134 (PMC12457402; doi:10.3389/fmed.2025.1606134)
Supplement: Supplementary file 3 [file Table_3.PDF]

Table S3. Patient's diastolic blood pressure (DBP) at different times (mmHg)

	Group PS	Group PE1	Group PE2	Group PE3	*P**
T0	81.53±11.33	76.37±11.94	78.03±12.91	82.17±11.10	0.180
T1	69.73±11.75	69.40±12.13	72.97±10.88	75.97±10.17	0.086
T2	70.73±12.50[a]	75.33±13.90[a, b]	78.73±11.64[b]	83.37±10.19[b]	0.001
T3	68.70±10.66[a]	67.20±11.93[a]	72.00±13.97[a, b]	79.67±9.00[b]	<0.001
T4	74.63±8.54[a, b]	71.83±7.87[a]	70.83±8.66[a]	78.63±6.84[b]	0.001

"ab" indicates the difference in diastolic blood pressure between the four groups at the same time. Groups sharing the same letter have no statistically significant difference ($P > 0.05$), while groups with different letters show a statistically significant difference ($P < 0.05$).
