# Supplementary material for: Safety and efficacy evaluation of low-dose of esketamine combined with propofol for painless gastroscopy: a single-center, randomized, double-blind, parallel controlled clinical trial
Source: Front Med (Lausanne). 2025 Sep 10;12:1606134. doi: 10.3389/fmed.2025.1606134 (PMC12457402; doi:10.3389/fmed.2025.1606134)
Supplement: Supplementary file 4 [file Table_4.PDF]

Table S4. Patient's SpO2 at different times (%)

	Group PS	Group PE1	Group PE2	Group PE3	*P**
T0	100[100,100]	100[100,100]	100[100,100]	100[100,100]	0.780
T1	100[100,100]	100[100,100]	100[100,100]	100[100,100]	0.797
T2	100[98.75,100]	100[100,100]	100[100,100]	100[100,100]	0.088
T3	100[99,100]	100[99,100]	100[100,100]	100[100,100]	0.172
T4	100[99.75,100]	100[98,100]	100[99,100]	99.5[98,100]	0.053
